# Scalp Eczema in Children

**Published:** 1907-05-18

**Authors:** 


### Dermatology.

### SCALP ECZEMA IN CHILDREN.

Considering the activity of the sebaceous glands in infancy and the ease with which micro-organisms flourish upon a seborrhœic basis, it is not surprising that eczema of the scalp, so-called, is such a common affection at this period of life. True eczema of the scalp in infants, apart from seborrhœa, is not an everyday occurrence, whereas a seborrhœic dermatitis affecting this region is constantly seen at a large skin clinic or by any busy practitioner. Cases of this sort divide themselves naturally into (*a*) the dry, scaly, and (*b*) the moist and crusted forms. The first type is met with as a fine, branny desquamation upon the scalp of quite young infants, especially in those with scanty hair. The disease encroaches upon the integument of the face and neck, and in these latter situations there may be a very little exudation of moisture. The term "pityriasis simplex capitis," sometimes applied to this condition, really describes a symptom only, and is not a true synonym for eczema. In older children the diagnosis from ringworm of the scalp is a matter of great importance, and in many cases it is quite impossible without a microscopical examination of a surface-scraping in potash solution to decide the question. An eczema is generally much more diffuse than tinea tonsurans, and in the latter a careful search with the aid of a lens will be almost sure to reveal the presence of at least one characteristic "stumpy" hair. Nevertheless, the one is frequently mistaken for the other, with sometimes disastrous results. The second, or crusted variety, is easier to diagnose, though some cases of inflammatory ringworm (kerion) simulate, at times, this form of eczema. When all crusts have been removed by appropriate treatment one or two suspicious hairs may then show the true nature of the case. Traumatism and local inoculation are the chief factors in producing a crusty eczema, and there are few conditions which look more formidable than a severe case of *Eczema crustosum* in a young infant.

With regard to the question of treatment, one is pretty sure to be met with the popular theory that the disease must on no account be cured too quickly in case it should "strike inwardly" and cause some internal malady of graver import, if not a fatal issue. There is no doubt that the risk of such results has been somewhat exaggerated, but, like most popular beliefs, this idea has some foundation in fact. In the so-called "eczema death" of infants, described by Feer, the "status lymphaticus" was present, *i.e.*, the lymphatic glands were generally enlarged throughout the body, as was also the spleen. A few cases have been reported in which signs and symptoms of true nephritis have supervened in the course of an impetiginous eczema of the scalp in children. The recent researches of Bernheim-Karrer have shown that a staphylococcic toxæmia has existed in such cases and has resulted in death from cardiac failure. The sudden suppression or disappearance of a scalp-eczema is not infrequently followed by convulsions, but when the ease with which these nerve-cell-explosions occur in infants is remembered too much significance need not be attached to this symptom. The case of Hahn is worth bearing in mind: an infant, after having had its scalp-eczema treated with an ointment containing litharge, died of convulsions believed to be saturnine, and a minute quantity of lead was recovered from the cerebral convolutions after death. Albuminuria is stated by Guinon and Pater to be not uncommon in these cases, and it is suggested that the fatal result is therefore due to renal insufficiency. Enough evidence is, therefore, forthcoming to inculcate a certain degree of caution in the treatment of eczema in young children.

If once the golden rule be recognised that where two ointments will cure an eruption the weaker is to be chosen, the practitioner will be on the right track. The very weakest of applications compatible with efficiency should always be employed. The oxides and the oleates of zinc and bismuth, five grains of each to the half-ounce of vaseline, with ten minims each of sweet almond and olive oils, make up a non-toxic yet remedial salve which may safely be applied to the scalp of an infant a few weeks old in cases of not too crusty eczema. When a complete mask of crusts presents itself two or three applications of the boracic-starch poultice, containing only ten grains of boric acid to two ounces of starch, will cause its effective removal, or, if this be impracticable, soaking the crusts with warm oiled linen rags is equally good. After the scabs have gone an ointment of zinc oxide, ten grs.; ammoniated mercury, three grs.; lanoline to one ounce, may be applied. No soap should be used, but the affected parts may be bathed with a very weak calamine lotion, or with a mixture of equal parts of cow's milk and warm water in which a little bran has been allowed to soak. The dressings should be lightly tied in place, and, in order to reduce the evil results of rubbing and chafing to a minimum, the child's hands may be muffled up or light cardboard splints may be applied to the arms, fixing the elbows. Strong antiseptics, especially coal-tar derivatives, are best avoided altogether in infantile scalp-eczema. In cases associated with pediculosis or impetigo, and these will generally be in older children, the amount of ammoniated mercury in the ointment may be increased to ten grs. to the ounce.

